# Counterconditioning following memory retrieval diminishes the reinstatement of appetitive memories in humans

**DOI:** 10.1038/s41598-019-45492-6

**Published:** 2019-06-25

**Authors:** Rani Gera, Segev Barak, Tom Schonberg

**Affiliations:** 1 Sagol School of Neuroscience, Tel Aviv University, 6997801 Tel Aviv, Israel; 2 School of Psychological Sciences, Tel Aviv University, 6997801 Tel Aviv, Israel; 3 Department of Neurobiology, The George S. Wise Faculty of Life Sciences, Tel Aviv University, 6997801 Tel Aviv, Israel

**Keywords:** Human behaviour, Consolidation

## Abstract

Appetitive memories play a crucial role in learning and behavior, but under certain circumstances, such memories become maladaptive and play a vital role in addiction and other psychopathologies. Recent scientific research has demonstrated that memories can be modified following their reactivation through memory retrieval in a process termed memory reconsolidation. Several nonpharmacological behavioral manipulations yielded mixed results in their capacity to alter maladaptive memories in humans. Here, we aimed to translate the promising findings observed in rodents to humans. We constructed a novel three-day procedure using aversive counterconditioning to alter appetitive memories after short memory retrieval. On the first day, we used appetitive conditioning to form appetitive memories. On the second day, we retrieved these appetitive memories in one group (Retrieval group) but not in a second group. Subsequently, all participants underwent counterconditioning. On the third day, we attempted to reinstate the appetitive memories from day one. We observed a significant reduction in the reinstatement of the original appetitive memory when counterconditioning was induced following memory retrieval. Here, we provide a novel human paradigm that models several memory processes and demonstrate memory attenuation when counterconditioned after its retrieval. This paradigm can be used to study complex appetitive memory dynamics, e.g., memory reconsolidation and its underlying brain mechanisms.

## Introduction

Appetitive associative memories play a crucial role in motivation, learning, behavior and decision-making^1^. Over the last two decades, it has been demonstrated that associative memories can be modified when manipulated during the process of ‘memory reconsolidation’ (for a review, see Lee *et al*.^2^). During this process, the reactivation of consolidated memories via their retrieval initiates a temporary labile state that lasts a few hours (“reconsolidation window”) during which memories are prone to modification before their restabilization^3,4^.

Interference with the reconsolidation process can potentially disrupt or update the original memory, which prevents its expression and, thus, holds significant clinical implications for disorders involving associative memories, such as addiction^5^. Despite the substantial clinical potential, to date, most studies involving humans have focused on the reconsolidation of aversive memories, whereas only a few studies have targeted the reconsolidation of appetitive memories. Several studies have employed pharmacological interventions during the reconsolidation of appetitive memories^6–10^ and yielded mixed results^11^. The use of beta adrenergic blockers (propranolol) yielded promising results^8,10^; however, these positive outcomes are not always consistent^6,9^. Importantly, such pharmacological agents may not be suitable for all populations (e.g., patients with bradycardia or some types of heart failure).

Previous studies in rodents have demonstrated the ability of behavioral manipulations (most commonly retrieval-extinction procedures) to affect the reconsolidation of associative memories^12–14^, and other studies have shown their capacity to affect the reconsolidation of human fear memory (e.g., see refs^15,16^). Recently, several studies applied behavioral manipulations (extinction, counterconditioning or cognitive reappraisal) during the reconsolidation of appetitive memories in humans^17–20^. Notably, these studies were conducted in individuals with substance use disorders^17,19^ or hazardous alcohol drinkers^18,20^. In these studies, the target memory existed prior to the study. The significance of these studies lies in their ability to demonstrate the effect of reconsolidation-based behavioral manipulations as a potential therapeutic treatment in clinical studies using pre-existing maladaptive memories. However, accumulation of knowledge regarding the basic mechanisms of appetitive memory dynamics based on these studies, is limited because these behavioral procedures were applied to patients. Specifically, conducting manipulations and fine-tuning experimental parameters typically cannot be applied to clinical populations. Moreover, normal and abnormal memory functions and their underlying neural mechanisms differ in healthy participants. Thus, while applying behavioral manipulations during reconsolidation in humans has shown encouraging potential as a therapeutic strategy for substance use disorders, currently, no basic scientific behavioral procedure is available to study the fundamental mechanisms underlying appetitive memory dynamics in healthy humans.

Therefore, here, we aimed to develop a novel human paradigm with the ability to disrupt the reconsolidation of appetitive memories in healthy participants. Establishing such a paradigm in which memory is formed and manipulated only under controlled conditions is essential for characterizing and tuning manipulations of memory dynamics. This basic scientific approach could enable investigations of the basic mechanisms of human appetitive memory. Additionally, establishing such a procedure could complement preclinical and clinical research in the field, which has already shown promising results^17–19^.

Recently, Goltseker *et al*.^21^ developed a novel behavioral paradigm in mice and demonstrated that administering aversive counterconditioning training following the retrieval of cocaine-associated memories abolished the reinstatement of cocaine seeking in mice. Das *et al*.^18^ successfully demonstrated the therapeutic potential of the retrieval-counterconditioning approach by attenuating measures related to pre-existing memories in human hazardous alcohol drinkers. In the present study, we aimed to translate the novel retrieval-counterconditioning animal model developed by Goltseker *et al*.^21^ into a novel basic scientific paradigm that first forms and then alters appetitive memories in healthy humans in the laboratory. Thus, we designed a behavioral procedure consisting of three stages. First, appetitive memories were formed in the laboratory; then, counterconditioning was applied, and finally, the memories were reinstated. We aimed to disrupt the reconsolidation of these memories by behavioral interference and therefore reactivated the memories prior to counterconditioning in one group, but not the other group, using a brief memory retrieval. We hypothesized (and preregistered our hypotheses and sample size) that this treatment will substantially reduce the reinstatement of these memories only in the retrieval group.

## Materials and Methods

### Participants

Ninety-six (63 females) healthy (nonclinical) participants participated in the experiment. The participants are considered healthy as they were recruited as widely accepted in studies involving the general population and did not undergo a formal clinical assessment. We aimed to obtain 25 valid participants that demonstrated adequate learning, based on certain criteria (see Experimental Design and Statistical Analysis section for details) in each of the following 2 groups: Retrieval and No-Retrieval (for the demographic characteristics of the 50 valid participants, see Table 1). Informed consent was obtained from all participants prior to participation in the experiment for monetary compensation (40 NIS = ~$12 USD per hour). The participants were informed that in addition to the hourly fee, they may gain or lose an additional monetary bonus. The participants were guaranteed that even if their losses exceed their gains as calculated from the beginning of the experiment, they would still receive at least the promised hourly rate. Therefore, their total compensation at each point could not be less than the hourly accumulated payment. All experimental protocols were approved by the ethics committee of Tel Aviv University. All methods were carried out in accordance with the relevant guidelines and regulations. Four participants were excluded due to software failures, and eight participants were excluded due to incompletion of the experiment. Thus, in total, 84 participants completed all parts of the procedure, and 50 of these participants (n = 25 per group) met our experimental inclusion criteria and were considered as valid participants.

**Table 1:** Demographic characteristics of the Retrieval and No-Retrieval groups.

Group	Gender (female)	Age mean (SD)	Dominant hand (right)
No-Retrieval (n = 25)	68%	25.92 (5.12)	92%
Retrieval (n = 25)	72%	23.72 (3.48)	88%
Total (n = 50)	70%	24.82 (4.52)	90%

*Note:* SD = standard deviation.

### Procedures

**Figure 1:**
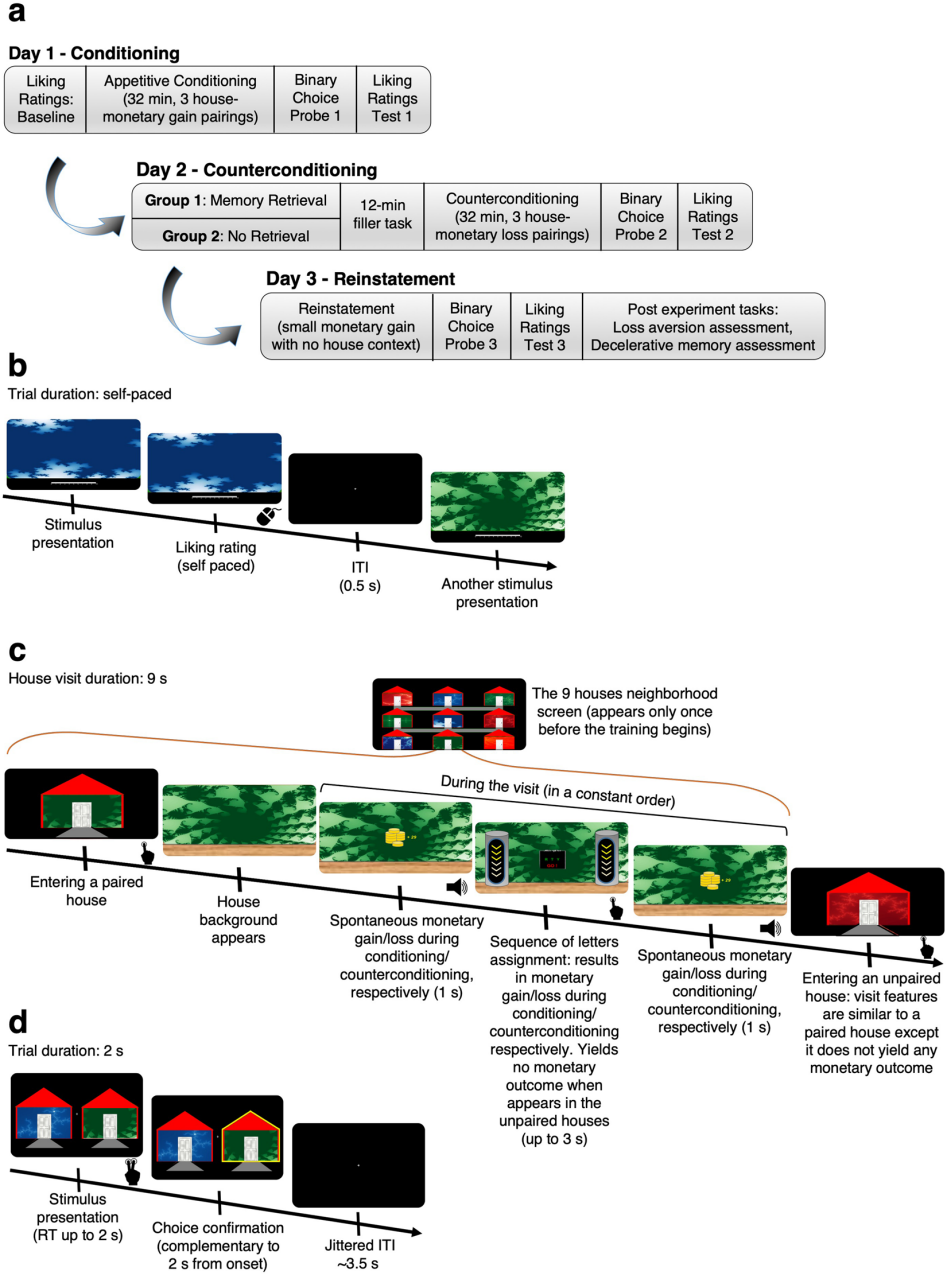
Experimental procedure outline and design. (**a**) General outline. The participants were subjected to three experimental stages over 3 consecutive days. (**b**) Liking ratings. The participants performed a baseline fractal liking ratings procedure. The same procedure was also performed following the conditioning, counterconditioning and reinstatement stages. (**c**) Simulated tour: conditioning/counterconditioning. Appetitive conditioning to simulated houses (CSs) was applied through a computerized simulated tour. In each of 20 blocks, the participants visited each of the 9 houses in the “neighborhood” once in a randomized order. The houses were divided into 3 equal-sized sets distinguished by color. One set (e.g., the green houses in this illustration) was paired with monetary gains through two Pavlovian rewards, and a reinforced instrumental task was performed between the rewards. This task was also used during the aversive counterconditioning on Day 2, in which the conditioned stimuli were paired with a monetary loss. A short version of this procedure was also used for the memory retrieval, in which the participants visited the paired houses, but did not gain or lose money. During reinstatement, the participants visited houses with no background (stimuli) and received a monetary gain. (**d**) Binary choice probe. During the probe sessions, the participants had to choose the houses they prefered to enter. Abbreviations: ITI, inter trial interval; RT, response time. The images in this figure were created by the first author (RG).

The experiment was programmed and performed in MATLAB R2014b (MathWorks, Natick, Massachusetts, USA) using Psychtoolbox 3^22,23^. The procedure consisted of the following three main stages: (1) appetitive conditioning, (2) aversive counterconditioning preceded by memory retrieval and (3) memory reinstatement. The experiment was carried out across three consecutive days. The participants were randomly assigned to the Retrieval and No-Retrieval groups. The overall workflow of the experimental procedures is illustrated in Fig. 1a. Briefly, on the first day (Day 1), we first measured the participants’ baseline liking ratings of different stimuli and assembled the conditioned stimuli (CSs) accordingly. Subsequently, the participants underwent appetitive conditioning. On the second day (Day 2), after retrieving the memory only in the Retrieval group, the participants in both groups underwent counterconditioning. On the third day (Day 3), we aimed to reinstate the memory formed through conditioning. The participants’ preferences and liking ratings were measured following the different stages of the task, and the changes in their preference toward and liking of the CSs were probed. We used computerized simulated houses as the CSs and monetary gains and losses as the appetitive and aversive unconditioned stimuli (UCSs), respectively. We chose monetary gains and losses to form relatively similar and comparable appetitive and aversive UCSs. The usage of gains and losses has been shown to effectively produce appetitive (e.g.^24,25^) and aversive (e.g.^26–28^) associations.

### Day one

#### Baseline fractal liking ratings

Upon arrival, the participants rated their liking of eighteen fractal art images (see https://osf.io/48uca/ for a source list) on a visual analogue scale (VAS) ranging from 0 to 10 (see Fig. 1b). The fractal collection comprised three color sets (red, green and blue), and each set consisted of six fractals. The fractal presentation order was randomized, and the task was self-paced. The purpose of this task was to obtain participants’ baseline liking ratings of the fractals and determine the stimuli assignment to CS+s and CS−s (see Supplemental Fig. S1 and Supplementary information).

#### Conditioning

The participants were informed that they will participate in a computerized simulated tour during which they will enter different houses (see Fig. 1c). In total, nine houses were designed with an identical generic wooden floor and were distinguished only by their background wallpaper. Each one of nine fractals (CSs) served as the background of one house. The participants were informed that in some houses, they will receive money, whereas in other houses, they will lose money. The purpose of this stage was to form an appetitive memory that could affect their subsequent choice behavior accordingly. The participants were not informed of the true goal of the task.

The task consisted of 20 blocks. In each block, the participants visited each of the nine houses once in a randomized order, and spent 9 seconds inside a house on each visit. Three houses from the same color set were assigned as CS+s and paired with a monetary gain. During each visit to these paired houses, a monetary reward (an average of 0.18 NIS, ~$0.05) was delivered twice; the participants were informed of the reward by a short animation of coins, and the sum gained was displayed accompanied by a winning casino-like sound (See Fig. 1c). In the other houses (CS−s), there were no monetary gains or losses.

In addition, we implemented an instrumental gaming-like sequence assignment that was scheduled to occur once during each house visit (see Fig. 1c and Supplementary Information). In the paired houses (CS+s), this assignment appeared between the two reward presentations. During the conditioning stage, success in an assignment in the paired houses (CS+s) yielded a monetary gain, while that in the unpaired houses (CS−s) yielded no outcome. While the participants were encouraged to respond as quickly as possible in the sequence assignments (see below), we did not explicitly direct them to notice, identify or act in any particular manner.

Thus, these sequence-pressing assignments were designed to increase the participants’ engagement and enhance the conditioning efficacy with an additional appetitive component paired with the paired houses (CS+s). The total duration of the conditioning stage was ~32 minutes including the transition between houses (excluding ~4 minutes of instructions and task demonstration).

To enhance the ease of learning, we used a constant presentation order within each 9-second visit to each paired house as follows: spontaneous reward (Pavlovian component), sequence assignment (instrumental component), and another spontaneous reward (Pavlovian component). To improve the realistic narrative of the simulated tour, we implemented several designated features (see Supplementary Information for a detailed description).

#### Binary choice probe on day 1

After conditioning, the participants performed a binary choice probe to measure their preferences toward the paired houses (CS+s) following conditioning. The participants were presented with two houses on each trial and allowed 2 seconds to choose the house they prefered to enter (see Fig. 1d). The participants were informed that on the final day of the experiment, one of their choices will be randomly chosen and that they will enter the house they had chosen on that particular trial and spend a few minutes inside that house. The probe phase included two blocks. During each block, each of the three CS+s was compared with all six CS−s for a total of eighteen comparisons (3*6). To avoid a mere-exposure effect^29^, we maintained an equal number of presentations of each CS throughout the entire procedure. Therefore, the probe included nine additional comparisons (3*3), each consisting of two different CS−s, i.e., one CS− from each of the two color sets assigned as CS−s. These comparisons were presented to the participants during each block. The trial order was randomized within each block.

#### Fractals liking ratings test on day 1

This test was identical to the baseline fractal liking ratings and was used to assess the changes in the liking of the stimuli that assembled the paired houses compared to the liking of the stimuli that assembled the unpaired houses following conditioning.

### Day two

#### Memory retrieval

The goal of this task was to reactivate the appetitive memories formed on day one to allow their subsequent alteration. Upon arrival, the participants in the Retrieval group performed a short simulated tour in the same neighborhood toured on day one. The task included two visits to each of the three paired houses (CS+s). The visits were similar to those on day one, except that (in contrast to day one) no money was earned during these visits. The instructions and presentation of the neighborhood screen were identical to the respective components of the conditioning stage performed on day one. The total duration of the retrieval task was ~1.5 minutes. For the participants in the No-Retrieval group, we did not employ a phase analogous to memory retrieval, similar to previous studies^15,30,31^.

#### Natural movie filler task

Following the completion of the Memory retrieval task by the Retrieval group, the participants watched a short nature video (~12 minutes including instructions) to provide a temporal separation between the memory retrieval and subsequent counterconditioning. Distraction from the original memory is important for inducing the destabilization of an existing memory and initiating reconsolidation rather than forming a new memory, which is similar to the case of extinction (reviewed in^32^). While watching the video, the participants were asked to answer several questions regarding its content (see Supplementary Information) to prevent cognitive engagement with the targeted memory. The interval between retrieval and counterconditioning was set to be similar in duration to previous studies using retrieval-extinction^12,15,17^ and retrieval-counterconditioning^18^ procedures. The participants in the No-Retrieval group watched the movie upon arrival without the memory retrieval phase. These participants completed this part at a nearby building with a different experimenter to avoid unintentional memory reactivation via the physical context^33^. Upon completion of the movie, the participants were instructed to return to the original testing room in the laboratory to continue the experiment.

#### Counterconditioning

The counterconditioning stage performed on day two was similar to the conditioning stage performed on day one, except for that the previously paired houses (CS+s) were paired with a monetary loss rather than a gain with equivalent amounts and contingency as day one. Consistently, all sequence assignments in the paired houses led to losses. The assignments in the unpaired houses remained neutral. The total duration of the counterconditioning stage was equivalent to that of the conditioning stage (~32 minutes). The purpose of this task was to associate the previously appetitive CS+s with an aversive stimulus to negatively affect the participants’ preference toward the CS+s.

#### Binary choice probe on day 2

This stage was identical to the previous Binary Choice Probe. This probe was used to confirm that counterconditioning led to a reduction in the preference toward the paired houses. We used a filler task that lasted ~5 minutes between both the conditioning and counterconditioning stages and the subsequent binary choice probe phase. This task was used to separate the learning manipulations from the subsequent tests (see Supplementary information).

#### Fractal liking ratings test on day 2

This test was identical to the previous fractal liking ratings test and was used to assess the relative changes in the liking of the stimuli that assembled the paired houses following counterconditioning.

### Day three

#### Reinstatement

Upon their arrival to the laboratory on day three, the participants in both groups performed a reinstatement task by re-exposure to the UCS^34^. This task was designed to reinstate the preference induced by the original appetitive associations created on day one. The participants were informed that they will take a short simulated tour similar to the preceding days, except for that the simulated houses will appear with no background wallpaper (i.e., without the fractals). During the task, the participants entered 6 no-background houses, all of which yielded a monetary gain similar to the paired houses on day one. Consistently, all sequence assignments led to wins. The total duration of the reinstatement task was ~1.5 minutes.

#### Binary choice probe on day 3

This stage was identical to the previous binary choice probes and was used to assess whether and the extent to which the reinstatement procedure recovered (i.e., increased) the preference toward the paired houses.

#### Fractal liking ratings test on day 3

This test was identical to the previous fractal liking ratings test and was used to assess the relative changes in the liking of the stimuli that assembled the paired houses following memory reinstatement.

### Memory and loss aversion measures

For a detailed description of the post-experimental tasks, see Supplementary Information. Briefly, we examined the participants’ loss aversion propensity using a modified version of a lottery choice task^35,36^ to verify that no group differences that may bias appetitive and aversive memory formation and manifestation existed. Then, we tested the participants’ recognition and contingency awareness of the stimuli used in the experiment, to estimate the potential interactions between our procedure and explicit memory indices and test for group differences. Finally, the participants were shown the actual resolved choices (via simulating an entrance to three of the houses they had chosen).

### Statistical analysis

#### Binary choice probe exclusion criteria

The binary choices between the CSs of each participant at the end of days one and two were used to determine whether to further analyze their results. Reinstatement could only be observed after successful conditioning and counterconditioning as manifested by preference toward the paired CSs on day one and its abolition on day two. Therefore, we only included data from participants who exhibited learning of these contingencies. We set the learning criteria as follows: (1) Successful conditioning was manifested as a paired choice proportion of 0.55 or higher. The rationale for this criterion was that we carefully designed the algorithm (see Supplemental Fig. S1) to assign the stimuli in a manner that was relatively immune to baseline preference bias and, thus, conceptually considered a choice proportion higher than 0.5 (chance level) an indication of learning. We added another 0.05 interval to define sufficient learning. In practice, the rate of learning of almost all participants that reached this learning criterion was much higher (see Results). (2) Successful counterconditioning was defined as a lower choice proportion compared to that following conditioning. Here, we did not use a specific value since a subjective reduction in individual preference rates (rather than an absolute value) is a suitable indicator of this effect. The participants who failed to meet both conditions were excluded from further analyses (see Results).

#### Binary choice analysis

We performed a mixed-effects logistic regression analysis between the groups (Retrieval and No-Retrieval) and stage (conditioning, counterconditioning, and reinstatement) with preference toward the CS+s as the dependent variable. As fixed effects, we entered the group and stage (with an interaction term) into the model. As a random effect, we included intercepts for the participants. Specifically, we were interested in the interaction between group (Retrieval and No-Retrieval) and the final two stages (counterconditioning and reinstatement). The binary choice outcome on each trial was marked as 1 or 0 according to the participant’s choice of the house paired with monetary gain/loss. Choice trials during which the participants failed to respond within the given two seconds were excluded from the analysis (35 observations, 0.65% of the data). On average, 107.3 (SD = 1.28) observations were obtained from each participant.

#### Liking ratings analysis

To assess the changes in the liking ratings, we measured the difference between the average liking ratings of the fractals that assembled the CS+s and the average ratings of the fractals that assembled the CS−s. Then, using a mixed-effects linear regression analysis, we regressed this index on the following variables: group (Retrieval and No-Retrieval) and stage (baseline ratings, conditioning, counterconditioning, and reinstatement) with an interaction term. As a random effect, we included intercepts for the participants. We were particularly interested in the interaction between the groups and the final two stages (counterconditioning and reinstatement).

#### Recognition memory

The recognition scores were calculated as the proportion of correctly recognizing whether a stimulus was presented during the experiment. Contingency awareness of the winning houses was defined as the proportion of correctly recognizing whether a stimulus included in the experiment was paired with a monetary gain. These memory indices and the loss aversion index were compared between the groups using an independent samples t-test. Contingency awareness of the losing houses was not measured in all participants due to a technical problem and, thus, was not analyzed. Two additional participants did not perform any part of the recognition and contingency awareness tasks.

All regression analyses were carried out using the lme4 package in R programming language (R Foundation for Statistical Computing, Vienna, Austria). The P-values of the linear regression analysis were estimated using the Satterthwaite approximation implemented in the lmerTest package.

#### Preregistration

We predetermined and preregistered our sample size, which was similar to that used in previous studies in the field (e.g.^15,17^).

The procedure, hypothesis, sample size, dependent variables and analysis methods of this study were preregistered (https://osf.io/un7y6/). To analyze the binary choice outcomes, we performed a mixed-effects logistic regression analysis as described above and not the ANOVA thatis listed in the preregistration. A regression analysis is appropriate for data with a binary dependent variable and, thereby, fits our binary choice data. To determine the changes in liking ratings outcomes, we performed a mixed-model ANOVA-equivalent mixed-effects linear regression.

#### Supplementary methods

See Supplementary methods for details regarding the apparatus, algorithm produced for the stimuli assignment, sequence assignment task implemented during the manipulation phases, features used to improve the task realism and a task used to separate the retrieval and counterconditioning phases.

## Results

Fifty participants (25 per group) of 84 (41 in the Retrieval group and 43 in the No-Retrieval group) met all learning criteria and were included in the analyses. Nineteen participants did not meet the learning criterion during the conditioning stage, while 15 participants did not meet the learning criterion during the counterconditioning stage. Thus, the successful acquisition rates during each learning stage were ~77%. The overall average choice proportion of paired houses (CS+s) among the participants included in the analysis was 0.89 following the conditioning phase and 0.37 following the counterconditioning phase (0.34 in the Retrieval group and 0.41 in the No-Retrieval group).

### Binary choice probe

**Figure 2:**
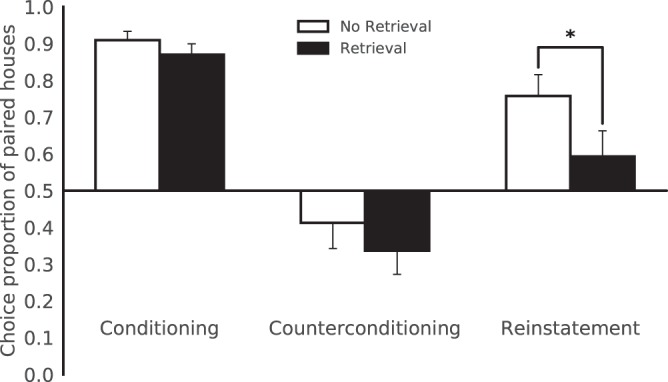
Retrieval prior to counterconditioning reduces the reinstatement of preferences. Mean proportion of trials during which the participants chose paired houses (CS+s) over unpaired houses (CS−s) following conditioning, counterconditioning and reinstatement. Chance level was 0.5, and error bars represent the standard error of the mean (SEM). *p < 0.05; n = 25 per group.

The mixed-effects logistic regression analysis of the preference toward the CSs (Table 2) showed a significant Group × Stage interaction (*χ*^2^(2) = 14.28, p = 0.0008). Following conditioning (Day 1), the participants in both groups preferred the CS+s (Fig. 2), and no difference was observed between the groups (p = 0.2542) as expected since no treatment differing between the groups was applied during this phase. Following counterconditioning (Day 2), the participants tended to avoid the CS+s (Fig. 2) as reflected by the Stage effects of conditioning and counterconditioning in both groups (Retrieval: OR = 25.59, 95% CI [19.32, 34.26], p < 2E-16; No-Retrieval: OR = 27.43, 95% CI [20.30, 37.54], p < 2E-16). Furthermore, as expected, we found no Group effect during the counterconditioning stage (p = 0.3036) or a specific interaction between Group and the first two stages (conditioning and counterconditioning) (p = 0.7456), consistent with the assumption that the retrieval manipulation does not affect counterconditioning.

**Table 2:** Logistic regression analysis of CS+ choices as explained by Stage, Group and their interaction.

	B	SE	OR	95% CI OR	p
Intercept	−0.36	0.30	0.70	[0.39, 1.25]	0.2275
Conditioning (day 1)	3.31	0.16	27.43	[20.19, 37.27]	0.0000
Reinstatement (day 3)	1.96	0.12	7.09	[5.56, 9.03]	0.0000
Retrieval Group	−0.44	0.43	0.64	[0.28, 1.49]	0.3032
Conditioning X Retrieval Group	−0.07	0.21	0.93	[0.61, 1.42]	0.7455
Reinstatement X Retrieval Group	−0.59	0.17	0.55	[0.4, 0.77]	0.0005

Counterconditioning (day 2) and the No-Retrieval group were used as the reference categories for the Stage and Group factors, respectively.

Re-exposure to the appetitive UCS (Day 3) reinstated the original preference in both groups, resulting in a tendency to prefer the CS+ houses again (Fig. 2). This finding was reflected in a Stage effect between counterconditioning and reinstatement showing greater preference toward the CS+ houses in both groups (Retrieval: OR = 3.93, 95% CI [3.15, 4.93], p < 2E-16; No-Retrieval: OR = 7.09, 95% CI [5.58, 9.06], p < 2E-16).

Retrieval vs. No-Retrieval: We found that in the Retrieval group, reinstatement was significantly weaker compared to that in the No-Retrieval group (Fig. 2) as supported by a Stage x Group interaction effect between counterconditioning and reinstatement (OR = 0.55, 95% CI [0.40, 0.77], p = 0.0005) and a Group effect during the reinstatement stage (OR = 0.36, 95% CI [0.15, 0.83], p = 0.0163). These results suggest that the retrieval of appetitive memories before their counterconditioning reduces their capacity to influence preferences.

### Liking ratings

We used a mixed-effects linear regression analysis to study the relationship among the learning stages (Stage factor), including the baseline, and memory retrieval manipulation conducted prior to the counterconditioning stage (Group factor), as manifested through changes in the liking of the fractals used to assemble the CS+s over the fractals that assembled the CS−s (Supplemental Table S1). In contrast to the altered preference toward the paired houses, we did not find a Stage x Group interaction (*χ*^2^(3) = 1.18, p = 0.7575) or a Group effect during any of the stages (p’s > 0.3617). Thus, we excluded the Group factor from the model and analyzed the Stage effect on liking across all participants. As illustrated in Supplemental Fig. S1 and consistent with the binary choice results, following conditioning (Day 1), the difference in the liking ratings between the CS+s and the CS−s was significantly increased (β = 0.52, t(147) = 7.428, p = 8.3E-12), and following counterconditioning, these ratings were reduced (Day 2) (β = −0.46, t(144) = −6.638, p = 5.73E-10) approximately to the baseline level. However, the reinstatement procedure (Day 3) did not reinstate the differences in liking observed following conditioning (β = 0.12, t(144) = 1.741, p = 0.0839), possibly suggesting that the limited delivery of the UCSs (a small quantity over a short duration) was not sufficient to reinstate the evaluation of the stimuli that assembled the CSs. Nevertheless, notably, analyzing the trends within each group without excluding the Group factor from the model yielded results corresponding to the general trend of the preference results. This finding is manifested as a significantly greater difference in the liking ratings between the CS+s and the CS−s following reinstatement relative to the baseline (β = 0.24, t(144) = 2.403, p = 0.0175) and a marginally significant difference relative to counterconditioning (β = 0.18, t(144) = 1.845, p = 0.0671) in the No-Retrieval group, indicating the potential recovery of the liking of the CS+s. In contrast, we found no differences among the equivalent stages in the Retrieval group (reinstatement vs. baseline: β = 0.11, t(144) = 1.153, p = 0.2510; reinstatement vs. counterconditioning: β = 0.06, t(144) = 0.601, p = 0.5490), suggesting that the original memory was not recovered following the reinstatement stage.

### Loss aversion

Two participants (one per group) were excluded from this analysis due to choice-inconsistency, namely, multiple switching points. No difference was found in loss aversion between the groups (t (46) = −0.93, p = 0.3564) with an average score of ~5.

### Recognition and contingency awareness of winning houses

In the recognition task, the participants in both groups correctly recognized ~97% of the stimuli with no difference between the groups (t (46) = −1.40, p = 0.1685). The contingency awareness of the winning houses in both groups was not as high (~76%) with no difference between the groups (t (46) = −0.09, p = 0.9299).

## Discussion

Using a novel behavioral basic science procedure performed in the laboratory, we demonstrate the updating of appetitive memories in healthy participants using aversive counterconditioning following memory retrieval. The recovery of an appetitive cue-monetary gain memory was diminished only when counterconditioning was performed after memory retrieval. The current paradigm is a successful translation of the retrieval-counterconditioning behavioral procedure previously used in animal models^21,37^. We suggest that this basic science paradigm provides a tool for future research investigating the underlying neural mechanisms of memory dynamics.

Our paradigm provides an approach complementary to previous, more clinically oriented attempts, demonstrating abilities to influence pre-existing alcohol memories in human hazardous drinkers^18^. Previous studies have employed behavioral manipulations targeting the reconsolidation of appetitive memories in clinical populations with substance use disorders. For example, Xue *et al*.^17^ showed that the retrieval-extinction procedure could reduce physiological and psychological measures of craving and relapse, which lasted for at least 6 months, in heroin addicts. Germeroth *et al*.^19^ used a similar paradigm and showed reduced cue-induced craving and relapse, which lasted for at least a month, in tobacco smokers. Kamboj and colleagues used either aversive-counterconditioning^18^ or cognitive reappraisal^20^ following memory retrieval and showed a reduction in alcohol craving and verbal fluency of positive alcohol-related words, respectively. Importantly, while these previous reports used post-retrieval behavioral manipulations to reduce craving in clinical settings, here, we developed a basic science procedure that forms (nondrug) appetitive memories in a fully controlled laboratory setting in healthy human participants. Therefore, our procedure offers a complementary approach that allows in-depth investigations of the fundamental mechanisms of human appetitive memory dynamics in healthy participants.

A plausible explanation for the reduced reinstatement we observed, is that the memory retrieval manipulation led to the destabilization of the appetitive cue-monetary gain memory. This memory destabilization allowed the aversive cue-monetary loss association to become incorporated into the appetitive memory trace during memory reconsolidation. Thus, the memory was altered and, consequently, reduced reinstatement. Goltseker *et al*.^21^ recently established a similar procedure in mice in which a cue-cocaine memory was first formed in a conditioned place preference (CPP) paradigm and then counterconditioned with lithium chloride-conditioned place aversion (CPA) following memory retrieval. Similar to our results, the retrieval-counterconditioning in mice prevented the reinstatement of appetitive cocaine CPP by a cocaine prime^21^. Critically, in the mouse study, counterconditioning performed 5 hours after or without memory retrieval failed to prevent reinstatement^21^. Moreover, this effect was long-lasting^21^. Thus, our findings and those reported in the mice study conducted by Goltseker *et al*.^21^ suggest that the retrieval-counterconditioning procedure involves memory reconsolidation mechanisms.

Aversive counterconditioning has been suggested to be more potent than extinction in suppressing appetitive memories^38,39^; however, its effect may be temporary^38,40,41^. Here, similar to Das *et al*. (2015), we show that when induced after a brief memory retrieval, aversive counterconditioning can suppress the reinstatement of appetitive memories, which is typically observed after counterconditioning. Interestingly, based on our memory recognition data, this procedure did not affect the ability to explicitly recognize the stimuli involved in the task or the awareness of the stimuli that yielded a monetary gain during conditioning. We also show that the effect was not due to differences in loss aversion between the groups, which could have potentially explained the differences in the reinstatement rates.

In addition to memory updating, the paradigm we constructed provides a valuable demonstration of the processes of counterconditioning and reinstatement. Previous human studies investigating aversive counterconditioning with laboratory-formed memories typically employed counterconditioning immediately following conditioning^39,42–44^, thus targeting short-term, preconsolidated memories. Under such conditions, it is possible that aversive counterconditioning interfered with the consolidation of appetitive memory^11^. In contrast, in the present study, a one-day interval between the conditioning and counterconditioning phases ensured that the appetitive memory was already consolidated at the time of counterconditioning.

Unlike the choice preference index, which was influenced by our procedure, the relative liking of the stimuli did not differ between the groups. As the effect associated with memory updating could only be manifested in our procedure as a relative decrement in reinstatement, we could not assess this effect with the liking index. In contrast to the preference index, the liking index was not contingent on the participants’ explicit monetary prospect considerations as the ratings were not associated with any future monetary outcome. Thus, it is possible that the intensity of the brief USC presentation was not sufficient for the manifestation of the behavioral reinstatement in the liking index. This could be attributed to the steadier nature of the acquired evaluation as manifested in its high resistance to extinction (for a review, see De Houwer *et al*.^45^).

Our study has several limitations. First, we decided not to include a control task in the No-Retrieval group because any different task could have been considered a confound and could have induced memory reactivation. However, this limitation could potentially be tested in future versions of the task. Second, we recruited healthy participants as widely accepted without a thorough clinical assessment. Future studies might consider performing such an assessment and/or performing this task in a clinical population to test its effects.

We excluded what seems to be many participants. We used a dual-stage learning criteria, i.e., for both the conditioning and counterconditioning stages. Approximately 23% of the participants were excluded following each of these stages (19 of 84 based on conditioning and 15 of the remaining 65 based on counterconditioning) due to these exclusion criteria. Thus, the overall proportion of exclusions (40%) stems from the conjunction of these criteria (in which large numbers of exclusions are anticipated^11^) as follows: only individuals who showed appetitive conditioning and subsequently successful counterconditioning were included in the final analyses. Furthermore, the relative complexity of appetitive conditioning^46^ may have hindered initial learning. However, in this study, both conditioning and counterconditioning yielded successful rates of ~77%, which is a satisfying rate compared to similar procedures employing dual-stage learning criteria^31,47^. Moreover, the participants who had learned exhibited high learning rates on average (see Results), suggesting that our manipulations are quite efficient and arguably justify the generalization of our results to the general population. Nevertheless, future steps aiming to further increase the proportion of learners, such as using multiple spaced sessions of conditioning and counterconditioning, could benefit the robustness of this procedure.

Finally, the reinstatement of the appetitive memories was reduced in the Retrieval group but not completely abolished. These findings are consistent with previous reports showing that the disruption of memory reconsolidation frequently leads to the suppression of the original behavior rather than its complete abolition^6,8,10,19^. It is possible that stronger aversive counterconditioning and/or more efficient memory retrieval (e.g., by expectancy violation) could have led to a stronger suppression or even complete abolition of the reinstatement effect. For example, a previous study involving humans used two consecutive days of retrieval-extinction training to decrease craving in abstinent heroin users^17^. Das *et al*. demonstrated that maximizing expectancy violation during memory retrieval before counterconditioning leads to a substantial reduction in measures of pre-existing alcohol memories in human hazardous drinkers^18,48^.

Over the past decade, several studies aimed to behaviorally interfere with the reconsolidation of associative memories. Although there is evidence of memory alteration, the updating process is seemingly complicated and has not been fully characterized to date. Our study, which was conducted under laboratory-controlled settings in a nonclinical sample, successfully demonstrates appetitive memory updating in humans by employing a novel retrieval-counterconditioning behavioral procedure. Therefore, it can be used for a wider inquiry of the basic mechanisms of human memory dynamics, relevant boundary conditions and the neural mechanisms involved in the process.

The procedure established here is characterized by a modular structure allowing researchers to selectively control and manipulate each stage and different components in it. Exploiting this property may enable the utilization of our procedure in studies investigating similar dynamics of aversive memories or other key mechanisms in the dynamics of associative memories. It can be used to compare different interventions, reinforcers and measurements, and study the neural mechanisms underlying normal (as opposed to pathologic) human memory processes. It should be noted that compared to real-life associative memories, the memories formed in this study were new and were not acquired through multiple spaced repetitions. Therefore, we cannot conclude that our manipulation could affect such real-life memories.

Finally, maladaptive processing of associative memories has been implicated in several neuropsychiatric disorders, including addiction^49,50^, OCD^51^, PTSD^52–54^ and phobias^52^. Specifically, the reinstatement capacity of associative memories is widely responsible for the persistence and relapsing nature of these pathologies, and for the transient effects of contemporary psychotherapy. For example, relapse in addiction is strongly related to the reinstatement of drug-associated memories^55^. Indeed, previous studies have demonstrated that memory disruption or updating can attenuate relapse and alleviate symptoms of related disorders^6,8,17,18,30,56–58^. However, others have failed to identify such effects using pharmacological (e.g.: in fear conditioning^59,60^, and in drug addiction^61,62^) or behavioral (e.g., see refs^20,31,63,64^) manipulations. The age and strength of the memory and the manipulation used to reactivate memory^8,65^ are all potential boundary conditions, which may complicate the translation of reconsolidation-disruption manipulations to human studies in clinical populations^66^. Therefore, our basic science procedure could be used as an interim translational step of reconsolidation-disruption manipulations in animals, hence allowing a smoother transitional process.

## Supplementary information

Supplementary Material

## Data Availability

All data and analysis codes are available on osf: https://osf.io/un7y6/.
